# Seroepidemiology and Molecular Epidemiology of Enterovirus 71 in Russia

**DOI:** 10.1371/journal.pone.0097404

**Published:** 2014-05-12

**Authors:** Ludmila V. Akhmadishina, Tatiana P. Eremeeva, Olga E. Trotsenko, Olga E. Ivanova, Mikhail I. Mikhailov, Alexander N. Lukashev

**Affiliations:** 1 Chumakov Institute of Poliomyelitis and Viral Encephalitides, Moscow, Russia; 2 Khabarovsk Institute of Epidemiology and Microbiology, Khabarovsk, Russia; Fondazione IRCCS Policlinico San Matteo, Italy

## Abstract

Enterovirus 71 (EV71) is an emerging human pathogen causing massive epidemics of hand, foot and mouth disease with severe neurological complications in Asia. EV71 also circulates in Europe, however it does not cause large outbreaks. The reason for distinct epidemiological patterns of EV71 infection in Europe and Asia and the risk of EV71 epidemic in Europe and Russia remain unknown. Seroepidemiology of EV71 and molecular epidemiology of occasional EV71 isolates were studied to explore circulation of EV71 in Russia. In six regions of Russian Federation, seroprevalence of EV71 in sera collected in 2008 ranged from 5% to 20% in children aged 1–2 years and from 19% to 83% in children aged 3–5 years. The seroprevalence among elder children was significantly higher (41–83% vs. 19–27%) in Asian regions of Russia. EV71 strains identified in Russia in 2001–2011 belonged to subtypes C1 and C2, while genotype C4 that was causing epidemics in Asia since 1998 emerged in 2009 and became dominant in 2013.

## Introduction

Enterovirus 71 (EV71) is one of 19 serotypes within the species *Human Enterovirus A* (HEV-A). EV71 is classified into three genotypes and 11 further subgenotypes: A, B1-B5, and C1-C5 [1]. Although EV71 infection is often asymptomatic, it can manifest as hand-foot-and-mouth disease (HFMD), severe encephalitis, and polio-like disease [2]. In Asia, EV71 has been causing extensive epidemics of HFMD and occasional neuroinfection, especially in the last 15 years (reviewed in [3]). Since 2008, regular epidemics of EV71 infection involving over a million cases have been reported in China [4]. In Europe, large outbreaks of polio-like disease caused by EV71 occurred in Bulgaria in 1975 and in Hungary in 1978. Thereafter, EV71 did not attract major attention until recently, when widespread asymptomatic circulation and detection from isolated neurological cases and minor outbreaks were reported from several European countries [5]–[8]. No major outbreaks have so far occurred in Europe. In Russia, no outbreaks of HFMD or neuroinfection caused by EV71 have been registered before 2013, and no studies of EV71 epidemiology have been carried out. Therefore, the disease profile and epidemiological situation differed strikingly between the adjacent regions of Russia and China during the 2008-2009 epidemic. The threat of EV71 epidemic urged investigation of EV71 circulation in Russia. A retrospective study conducted in Taiwan following the epidemic of 1998 reported low rate of seroconversion against EV71 in infants aged six months to three years prior to the epidemic and implied it as a potential risk factor of the epidemic [9], and at that time seroepidemiology could be seen as a good measure to predict EV71 epidemics. Here we studied seroepidemiology of EV71 in six regions of Russia and summarized occasional isolations of EV71 over 13 years.

## Materials and Methods

**Virus isolation** from fecal samples was performed by World Health Organization (WHO) Regional Reference Laboratory in Moscow and the collaborating WHO regional laboratories according to the WHO polio surveillance protocol [10] in RD (rhabdomyosarcoma) cell culture. Samples were initially collected for polio surveillance mainly from children with acute flaccid paralysis and contacts, not for systematic screening for EV71. The virus isolates were identified in neutralization test with the panel of antisera (National Institute for Public Health and the Environment, Bilthoven, Netherlands). The virus type was then confirmed by sequencing of partial virion protein 1 (VP1) protein encoding genome region [11].

**Neutralization test** was performed in RD cell culture. Serial two-fold dilutions of serum, starting with 1∶8, were incubated with 50–100 50% tissue culture infectious doses (TCID_50_) of the virus for one hour at 37°C and added to cell culture in 96-well plates. Two wells were used for each titration point. Cytopathic effect was accounted 4 days after infection. Antibody titer of individual sera was expressed as a reciprocal of a dilution that completely neutralized infectivity. Neutralizing titer of 8 and higher was considered positive.

**Figure 1:**
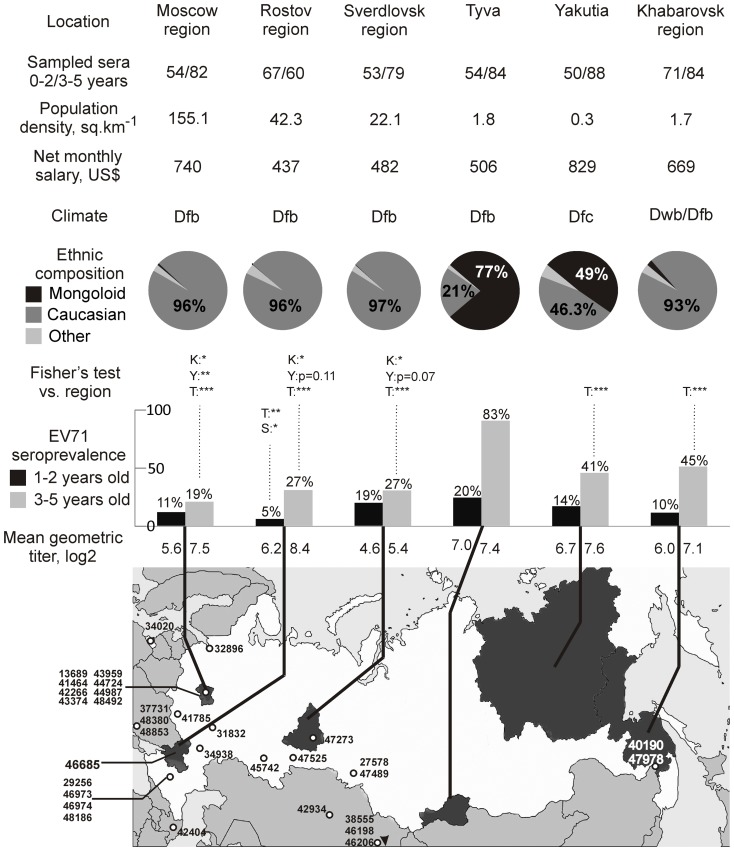
Sampling territories (shaded on the map), the number of sampled sera, sociogeographic conditions in the study regions, prevalence and mean geometric titers of antibodies to EV71 in children aged 1–2 and 3–5 years. Social and economic status of the regions is provided according to the official census and statistical data. Climate zone is indicated in accordance with Köppen classification. Dfb, warm summer continental, even annual rainfall; Dfc, continental subarctic, even annual rainfall; Dwb, warm summer continental, low winter rainfall. Isolations sites of EV71 strains presented in Fig. 2 are indicated with empty circles. Significance of Fisher's exact test comparing seroprevalence in younger and elder age groups is indicated above the bars of the seroprevalence graph; comparison regions are indicated by first letters. Only significant values are shown; * indicates p<0.05, ** p<0.01, *** p<0.001.

**Serum samples** were collected from healthy individuals in six regions of Russia: Moscow region (excluding the city of Moscow), Rostov region, Sverdlovsk region, Yakutia, Tyva and Khabarovsk region. Number of samples from each region is provided in Figure 1. The subjects were randomly sampled by computer-assisted selection from an address book in 2007–2008 for a study on hepatitis A seroprevalence. Only sex and age of study subjects were recorded, information on ethnic background and travel history of individuals were not available. Written informed consent was obtained from all subjects or their legal representatives. The study was approved by the Ethical Committee of the Chumakov Institute of Poliomyelitis and Viral Encephalitides. A total of 831 sera from healthy children aged 1–5 years and from 30 adults were used for this study. The children were further divided into two groups of 1–2 and 3–5 years of age because the latter corresponds to a common age of kindergarten admission in Russia.

Information on social and economic factors was derived from the official 2006 census data [12], climate zones in the sampling regions according to were provided according to the updated Köppen classification [13].

Statistical significance of seroprevalence differences was estimated using Fisher's exact test. Mean geometric titers were calculated with Mann-Whitney test. Correlation was estimated with Spearman's test.

### Phylogenetic analysis

Complete VP1 genome region sequences (891 nt) of EV71 isolates were identified by Sanger sequencing after PCR with oligonucleotides VP3F (GCG CCC AAY ACA GCY TAY ATA ATA GCA) and 2AR (TGG CGA GRT GRC GRT TRA CCA CTC T). Out of over 3000 EV71 VP1 sequences available in Genbank, we selected all sequences of genotype C that differed by at least 1% of nucleotide sequence (330 sequences) Phylogenetic analysis was done using Bayesian likelihood-based algorithm implemented in Beast version 1.7.5 [14]. The SRD06 substitution model optimized for coding sequences [15] was used with a relaxed exponential clock setting, which was better than the strict clock and the relaxed lognormal clock upon Bayes factor test (Log10 Bayes factors >10). The population prior had no significant effect on the analysis; therefore the constant population prior was used. Each analysis was run over 100 million generations and trees were sampled every 10,000 generations, resulting in 10,000 trees. Trees were annotated with TreeAnnotator v.1.4.8 using a burn-in of 1,000 trees and visualized with FigTree v.1.3.1 (http://tree.bio.ed.ac.uk/software/figtree/).

Genbank accession numbers of Russian EV71 isolates are JQ973699-05, KJ645794-814, KC879485-527.

## Results

### Selection of the virus strain

EV71 prototype strain BrCr, which is commonly available from virus collections, belongs to the extinct genotype A, while most circulating viruses belong to genotype C. An EV71 strain isolated in Moscow in 2001 (strain 13689, genotype C2, see below) was the only contemporary strain available at the beginning of the study in 2008. It was compared in a preliminary neutralization test experiment with the prototype strain BrCr to select the most suitable virus for the main study. A panel of 30 sera from children aged 3–9 years and 20 sera from adults aged 32–73 years collected in 2008 in Khabarovsk region (Far East of Russia) was used because this region borders the epidemic area and was therefore of prime interest for the study. The same sera were positive with both viruses. The number of positive sera was 17/30 (57%) in children and 19/20 (95%) in adults. The mean geometric neutralizing titers against strain BrCr were somewhat higher in adults (45 vs. 18, Mann Whitney P = 0.006), while in children neutralizing titers were slightly higher against the contemporary genotype C2 strain 13689 (145 compared to 67, Mann Whitney P = 0.07). In 5 out of 17 seropositive children the antibody titer against strain 13689 was significantly (4–16 times) higher than against strain BrCr. As infants are the most susceptible to EV71 infection and were the target group of the main study, strain 13689 was used for further neutralization tests. The higher neutralizing titers against genotype A strain BrCr in adults suggest that the viruses that elicited immune response were antigenically distinct from genotype C and probably represented genotype A.

**Seroprevalence of EV71 infection** was investigated among children below 5 years old in six regions of Russia (Fig. 1). The prevalence of EV71 antibodies in the younger age group varied from 5% to 20% in different regions without any obvious pattern. In the older age group, the fraction of children that had encountered EV71 was higher (19–83%). In two areas (Moscow and Sverdlovsk regions) the difference between the age groups was not statistically significant (p>0.05, Fisher's test), in other four regions it was significant (p<0.05, Fisher's test). Interestingly, mean geometric titers of antibodies against EV71 were consistently higher in elder children (Fig. 1), although this difference was not statistically significant.

### Possible factors linked to high EV71 seroprevalence

In children aged 3-5 years EV71 seroprevalence differed significantly between the regions of Russia. There was no apparent link between the EV71 seroprevalence and population density or income level in a region (Fig. 1), but the general statistics do not necessarily reflect exact conditions at the point of sampling. The climate in all study regions is cold continental, and minor variations of climate could not explain significant difference in EV71 seroprevalence. EV71 seroprevalence was notably lower in the sampling regions located in the European part of Russia than in the Asian regions (19–27% vs. 41–83%). Khabarovsk Region and Tyva have frequent trans-border contacts with China and Mongolia, respectively. Moreover, Amur, the major river that flows through the main cities of the Khabarovsk region, receives runoff from China and could promote spread of enteroviruses. However, intensity of travel to South-East Asia was not universally correlated to EV71 seroprevalence. The highest number of regular flights to China and East Asia originate from Moscow airports (on average 18 flights per day), but this did not result in elevated EV71 seroprevalence. Other study regions have on average one or less flight to endemic regions per day. Yakutia, with its 41% EV71 seroprevalence rate, also has about one direct flight to South-East Asia per day and very limited possibilities for land travel.

The proportion of EV71 seropositive children in the older age group could be also linked to the prevalent ethnic background in the region. EV71 seroprevalence was high in Tyva and Yakutia, two regions with 77% and 48% population of Asian ethnic background, respectively. Seroprevalence among children aged 3–5 years correlated with the proportion of Asian ethnic background in the region (Spearman r = 0.89, P<0.05). Importantly, social, religious, and food habits differ considerably between the two regions with a high fraction of Asian population.

### Phylogenetic analysis of EV71 isolated in Russia in 2001–2013

Out of over 4000 non-polio enterovirus cell culture isolates that were submitted to the WHO Regional Reference Laboratory in Moscow in 2000-2013 by the WHO polio surveillance centers, only 48 were identified as EV71. Twelve viruses were identical to other isolates, 3 were not analyzed for technical reasons. Three viruses belonged to subtype C1, and 12 were subtype C2. Importantly, Russian C2 subtype isolates included a strain termed 40190, which was isolated in 2010 in Khabarovsk, 10 kilometers from the Chinese border, during an EV71 outbreak in China caused by C4 subtype [4]. 18 viruses represented genotype C4. First isolation of genotype C4 in Russia dated to 2009. In 2013, genotype C4 was the most common in Russia, accounting for 11 of 14 isolations.

**Figure 2:**
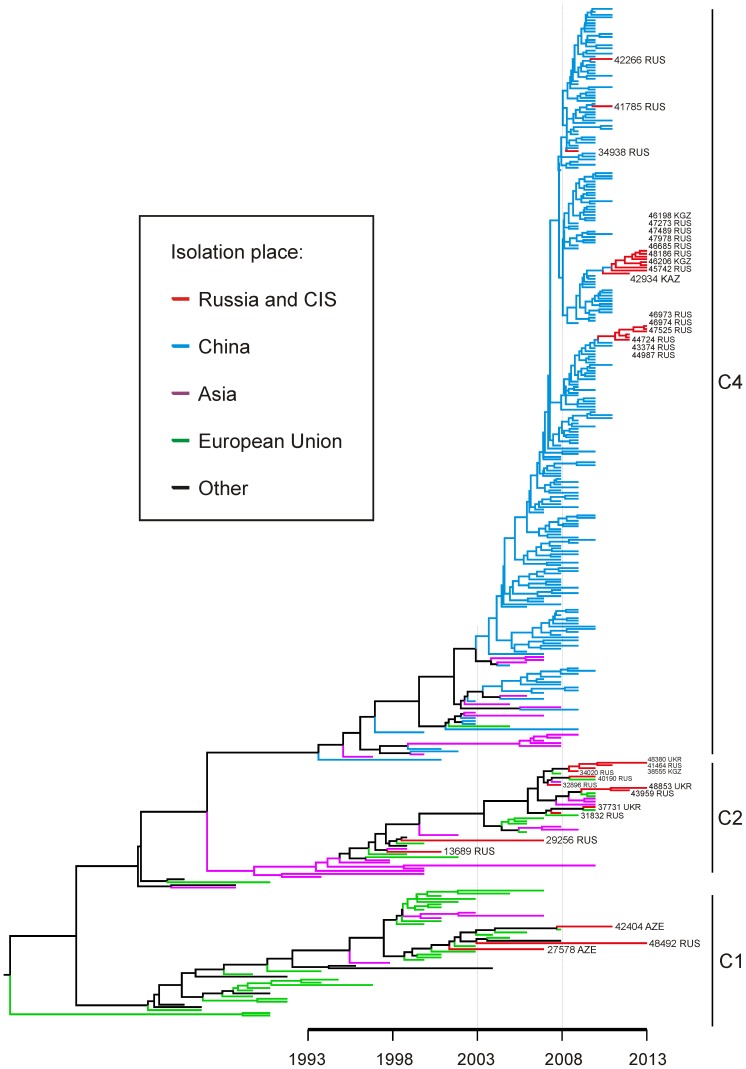
Phylogenetic tree of EV71 isolates from Russia and CIS (indicated in bold) and all GenBank sequences of EV71 genotype C that differ by over 1%. For Russian/CIS sequences, the isolate number is indicated, which refers to the sampling location indicated in Fig. 1. The tree was created using the complete VP1 genome region (891 nucleotides) and the Bayesian Markov Chain Monte Carlo algorithm implemented in Beast 1.7.5. Scale bar indicates time. Branches are colored according to isolation region indicated in the legend.

Phylogenetic analysis indicated that in many cases Russian isolates did not group together, or grouped reliably with different reference sequences (Fig. 2). Such grouping pattern is compatible with multiple introductions of virus to/from Russia; however it is not possible to establish the exact number and direction of such transfers because of low posterior probability support on some nodes.

## Discussion

Seroprevalence of EV71 varies significantly between different regions of the world, and even between samples from the same region. Few studies were conducted in countries that did not have epidemics of neuroinfection. In Germany, one study reported seroprevalence of 12% at age of 1–4 years [16], while another publication reported seroprevalence of 27.3% at 0–3 years and 45.6% at 3–6-years [17]. These seroprevalence rates were not obviously lower than in the countries that experience massive epidemics of EV71 infection. In China, retrospective analysis revealed 40% seroprevalence of EV71 before the 2008 outbreak [18]. In Taiwan, EV71 seroprevalence in children was 22–36% before the 1998 epidemic and 24–42% after the epidemic [19], [20]. In Singapore, which is located in the endemic region, but had only limited outbreaks, seroprevalence among children aged 3–5 years was 30% [21].

Seroprevalence of EV71 in Russia among infants aged 1–2 years ranged from 5% to 20% without an obvious pattern. In children aged 3–5 years seroprevalence of EV71 was expectedly higher and differed strikingly between regions of Russia. In three European regions, it was 19–27%. Significantly higher seroprevalence of EV71 was found in the Asian regions of Russia. In Yakutia and Khabarovsk region, it was 41% and 45%, respectively, about as high as in countries of South-East Asia that reported epidemics in the last decade. In one Russian region, Tyva the seroprevalence of EV71 (83%) was the highest ever reported in this age group. Comparable numbers (85%) have only been reported among elder children (5–15 years) in Vietnam [22]. Proximity of Asian regions to the “endemic” countries could explain this pattern, but only in Khabarovsk and Tyva. In addition, there is no evidence that overt morbidity in endemic regions correlates with the seroprevalence. Yakutia had a 40% EV71 seroprevalence despite very limited opportunities for virus transmission from Asia. Host genetics, reflected in the prevalent Asian ethnic background, could also affect the EV71 seroprevalence in Yakutia ant Tyva.

Genetic host factors have been implicated in EV71 epidemiology previously. Indeed, despite comparable seroprevalence levels, only Asian countries are repeatedly hit by outbreaks of overt disease. It has also been shown that genetic background of patients affects severity of EV71 infection. One study associated HLA-A33 genotype and several other genetic polymorphisms were significantly associated with susceptibility to EV71 [23]. A CTLA-4 polymorphism has been also associated with severity of EV71 infection [24], however this finding was not supported by other studies [23]. The role of host genetics in susceptibility to EV71 infection was also suggested in a study on EV71 seroepidemiology in Singapore, which reported up to five-fold differences in HFMD incidence in groups of patients of Asian versus Caucasian ethnicity [25]. However, as socioeconomic status and sanitary habits also differed between these groups, it was not possible to establish the cause of different morbidity. In Russia, higher EV71 seroprevalence was found in regions with prevalent Asian ethnic background. Unfortunately, samples used in this work were collected without information on ethnic background, and further studies are required to investigate the role of host genetic background on EV71 infection, both symptomatic and asymptomatic.

Overall, the extreme variation of EV71 seroprevalence between regions of Russia was not correlated to incidence of reported EV71 neuroinfection, which was zero in all regions before 2013. Therefore, seroepidemiology is a poor predictor of EV71 epidemic risk.

Systematic enterovirus surveillance in Russia functions only in few regions, and does not aim to investigate viruses beyond type identification. WHO polio surveillance network is responsible mainly for acute flaccid paralysis surveillance; however it also provides a good coverage also for non-polio enteroviruses. We analyzed molecular epidemiology of EV71 strains isolated by WHO polio surveillance network in 2001-2013. Importantly, although most of these viruses were isolated from children with neurological manifestations, this does not allow implicating them as the causative agents of these AFP cases because hundreds of other enteroviruses are routinely isolated from non-viral AFP cases each year in the polio surveillance network.

Before 2011, genotypes C1 and C2 were more prevalent in Russia. All isolates of these genotypes had the closest relatives among viruses identified in Europe, and these two subtypes were also the most common EV71 subtypes detected in European countries [6]-[8]. Subtype C4 was occasionally isolated in Europe, but it constituted only a minor fraction of all EV71 detections [7], [17]. Subtype C4 was first detected in Russia in 2009 and became predominant in 2013. In China, genotype C4 caused epidemics in 2008, 2009, and 2010, while genotypes C1 and C2 were rarely detected [4]. Spread of genotype C4 to other regions was anticipated [6], however in Russia is was not followed by epidemic of EV71 neuroinfection up to 2013. Phylogenetic analysis of VP1 genome region suggested multiple introductions of the virus to Russia, but did not allow establishing the exact number and direction of EV71 transfers, because the sample of sequences available in Genbank (especially for genotypes other than C4) was not sufficient (both by the size and by the geographic coverage) to trace epidemiology of EV71 with sufficient resolution.
